# By-Laws Buffalo Medical Library Association

**Published:** 1883-03

**Authors:** 


### By-Laws Buffalo Medical Library Association.

### ARTICLE I.

*Regular Members.*

Sec. 1. Any member of Erie County Medical Society may, upon approval of the Board of Directors, and by signing the Constitution and By-Laws, and paying his annual dues, become an active member of the Association.

§ 2. The representatives of the United States Army or Navy and Marine Hospital Service, stationed at Buffalo, may become active members under the same conditions as those contained in Section 1 of this Article.

§ 3. Any person may, on approval of the Board of Directors, and on the payment of *three dollars* ($3.00), become a subscribing member and have the use of the reading room; but subscribing members shall not have the privilege of taking out books or journals, or of holding office or voting at the meetings of the Association.

§ 4. The annual dues of the Association shall be five dollars ($5.00), payable in advance. They shall date from January 1st, and the dues of new members, joining at other times of the year, shall be at the rate of forty-two cents monthly, up to the first of January next ensuing.

§ 5. Any active member may commute his dues by the payment of one hundred dollars ($100.00) at one time, for which he shall receive a certificate of life membership, signed by the President and Secretary. Such payment shall exempt him from the payment of any further dues to the Association. All moneys received for life membership shall be invested in a manner approved by the Board of Directors and shall be known as the *Permanent Fund*, and the interest only of such fund shall be used for current expenses of the Association.

§ 6. If any member shall fail to pay his annual dues, within one month after due notice is personally served from the Board of Directors, he shall be liable to forfeit his membership on vote of the Board of Directors.

§ 7. Members whose dues are six months in arrears, shall not be qualified to vote or hold office.

§ 8. Members shall be considered liable for dues until a formal resignation is received and accepted by the Board of Directors. No resignation shall be accepted until the member's dues are paid to date or unless specially remitted by the Board.

### ARTICLE II.

*Officers.*

Sec. 1. The President shall preside at the meetings of the Association and shall be *ex-officio* President of the Board of Directors.

§ 2. The President, at the request of the Board of Directors, or at the request of five members of the Association, stating the reason therefor, shall call a meeting of the members of the Association for the transaction of special business, sufficient notice thereof having been served personally upon all members of the Association.

§ 3. The Vice-President shall in the absence of the President perform all the duties of his office.

§ 4. The Secretary shall keep an accurate record of all transactions of the Association and of the Board of Directors.

§ 5. The Treasurer shall receive all dues and donations of money, pay all drafts on him when signed by the Secretary and countersigned by the President, and keep a regular account of the financial concerns of the Association, an abstract of which, accompanied by satisfactory vouchers, he shall exhibit at each annual meeting.

§ 6. The Librarian shall have charge of all publications belonging to the Association and of all other property belonging to it not in the hands of other persons. He shall have the management of the library and reading-room, furnish a report of the condition of the library at the annual meeting and shall also be *ex-officio* Corresponding Secretary of the Association.

§ 7. The Trustees shall be divided by lot into two classes of two each, who shall hold office as follows, viz.: The first class for one year and the second class for two years. One class shall be regularly elected at the annual meeting the Association, in December of each year, to hold office for two years.

### ARTICLE III.

### *Meetings.*

SEC. 1. The Board of Directors shall meet on the third Tuesdays of May, November and December, and at other times at the call of the President.

Notices of a meeting shall be issued by the Secretary at least two days prior to such meeting. Five members shall constitute a quorum. At the December meeting the standing committees for the year shall be appointed, and the amount determined for the Committee on Journals and the Committee on Books to expend during the ensuing year.

§ 2. There shall be an annual meeting of the Association on the first Friday in December, when an election of officers for the ensuing year shall be held by ballot: the reports of the Board of Directors, Treasurer and Librarian read, and such other business be transacted as may be presented.

Twelve members shall constitute a quorum.

§ 3. The President shall at the November meeting of the Board of Directors appoint an Auditing Committee of three members of the Association, not members of the Board of Directors, to examine the accounts and vouchers of the Treasurer and submit a report on the same at the annual meeting of the Association.

§ 4. In case of any vacancy occuring in the Board of Directors by death, resignation or removal, the Board shall have power to fill such vacancies.

§ 5. At least three days previous to the annual meeting notice of the same shall be issued to every member.

ARTICLE IV.

*Library.*

SEC. 1. The Board of Directors shall have power to make proper provisions and regulations for the preservation, management and direction of the library and reading-room, and for the purchase of all books and periodicals.

§ 2. A committee of two shall be appointed by the Board of Directors, at its November meeting, to examine the books and papers of the Association, and report thereon at the annual meeting.

ARTICLE V.

*Committees.*

SEC. 1. The standing committees shall consist of a Committee on Books and a Committee on Journals; each committee to consist of three members of the Board of Directors. The Librarian of the Association shall be, *ex-officio*, one of the members of each committee.

ARTICLE VI.

*Amendments.*

SEC. 1. These by-laws may be amended by a two-thirds vote at any regular meeting of the Board of Directors, notice in writing having been given at a previous meeting.

